# Genetic variability of hepatitis B virus in acute and in different phases of chronic infection in Brazil

**DOI:** 10.1038/s41598-024-60900-2

**Published:** 2024-05-10

**Authors:** Barbara Vieira Lago, Moyra Machado Portilho, Vinicius Motta Mello, Paulo Sergio Fonseca De Sousa, Giovana Paula Angelice, Bianca Cristina Leires Marques, Larissa Tropiano da Silva Andrade, Vanessa Alves Marques, Lia Laura Lewis-Ximenez, Francisco Campello do Amaral Mello, Livia Melo Villar

**Affiliations:** 1 Laboratório de Hepatites Virais, Instituto Oswaldo Cruz, Fiocruz, Hélio and Peggy Pereira Pavillion, Ground Floor, Office B09, FIOCRUZ Av. Brasil, 4365, Manguinhos, Rio de Janeiro, RJ 210360-040 Brazil; 2 Instituto de Tecnologia em Imunobiológicos (Bio-Manguinhos), Fiocruz, Rio de Janeiro, Brazil

**Keywords:** Evolution, Microbiology, Molecular biology, Diseases

## Abstract

The selection pressure imposed by the host immune system impacts on hepatitis B virus (HBV) variability. This study evaluates HBV genetic diversity, nucleos(t)ide analogs resistance and HBsAg escape mutations in HBV patients under distinct selective pressures. One hundred and thirteen individuals in different phases of HBV infection were included: 13 HBeAg-positive chronic infection, 9 HBeAg-positive chronic hepatitis, 47 HBeAg-negative chronic infection (ENI), 29 HBeAg-negative chronic hepatitis (ENH) and 15 acute infected individuals. Samples were PCR amplified, sequenced and genetically analyzed for the overlapping POL/S genes. Most HBV carriers presented genotype A (84/113; 74.3%), subgenotype A1 (67/84; 79.7%), irrespective of group, followed by genotypes D (20/113; 17.7%), F (8/113; 7.1%) and E (1/113; 0.9%). Clinically relevant mutations in polymerase (tL180M/M204V) and in the Major Hydrophilic Region of HBsAg (sY100C, T118A/M, sM133T, sD144A and sG145R) were observed. Our findings, however, indicated that most polymorphic sites were located in the cytosolic loops (CYL1-2) and transmembrane domain 4 (TMD4) of HBsAg. Lower viral loads and higher HBV genetic diversity were observed in ENI and ENH groups (*p* < 0.001), suggesting that these groups are subjected to a higher selective pressure. Our results provide information on the molecular characteristics of HBV in a diverse clinical setting, and may guide future studies on the balance of HBV quasispecies at different stages of infection.

## Introduction

Hepatitis B virus (HBV) is a hepatotropic pathogen that indirectly causes liver disease of variable severity^1^. Due to the interplay between the virus and the host immune system, HBV infection may result in different clinical outcomes, ranging from acute (with or without clinical manifestations), to chronic or fulminant hepatitis^2^.

HBV is replicated by an error-prone polymerase through an RNA intermediate, leading its classification into 10 genotypes, several subgenotypes and diverse intra host viral variants called *quasispecies*^3–6^.

Upon transmission, distinct evolutionary events shape the architecture of the HBV *quasispecies*. During acute hepatitis B (AHB), both innate and adaptive immunity are triggered. In the first stages of infection, the bottleneck effect drives HBV evolution^7^. Under selective pressure from the immune system of a new host, HBV isolates are subjected to founder effect, selecting viral variants that are able to evade the immune pressure of this new environment, thus establishing infection^8,9^.

In chronic hepatitis B (CHB), HBV *quasispecies* balance is also a dynamic process, reflecting the interplay between HBV replication and the host immune system defense mechanisms^2^. Under prolonged immune pressure, viral subpopulations fluctuate over time^4,7,10^. Thus, in both acute and chronic infection, viral evolution is shaped by distinct immunological events, resulting in diverse outcomes^6,7^. CHB can be divided in five clinical phases, according to virus replication status—reflected by the presence/absence of HBeAg—and liver inflammatory disease: (i) HBeAg-positive chronic infection, (ii) HBeAg-positive chronic hepatitis, (iii) HBeAg-negative chronic infection, (iv) HBeAg-negative chronic hepatitis and (v) HBsAg-negative phase (HBV occult infection)^2^.

It has been postulated that amino acid mutations in the Major Hydrophilic Region (MHR) of the envelope gene (HBsAg) may be responsible for changes in viral antigenicity, affecting not only the immune response but also the disease outcome in patients carrying clinically relevant variants^6,11^. These mutations may impact on drug resistance, response to vaccination, diagnosis and may play a role in the progression to HBV-associated liver disease^5,6^. Moreover, substitutions in HBsAg may impair virion secretion, HBsAg formation ^12–14^ and can affect HBsAg serum levels by modulating the secretion of the surface proteins^15–17^. Inconstancies in HBsAg secretion may also have consequences in HBV detection by commercial assays^14^. In addition, high HBsAg genetic diversity has been observed in patients with advanced liver disease ^5,18^.

Despite has been demonstrated that HBV variability may impact in the natural history of HBV infection^6,10,19^, few studies have investigated HBV diversity under different selective pressures. The association between HBV diversity and clinical outcomes in both acute and in the different phases of chronic infection remain unclear. In this study we analyzed HBV genetic variability, drug resistance and immune escape mutations in the overlapped HBV polymerase/surface genes in HBV carriers in different stages of hepatitis B infection.

## Results

### Study population

One hundred and thirteen individuals in different phases of hepatitis B infection were evaluated in this study. Most of them were male (60/113; 53.1%) and overall mean age was 44.6 ± 14.4 years. Most samples were collected at baseline however, 13 individuals reported previous HBV treatment. Demographic and virological features of the 113 subjects according to HBV infection phase are stated in Table 1.

**Table 1:** Demographic and virological features according to HBV infection phase.

HBV groups	Sex assigned at birth		Age (years)	Therapy*	Coinfections
			mean ± SD	n/N (%)	Infection (n)
	F	M			
Acute	7	8	42.2 ± 9.9	0/15 (0.0)	HCV (1)
EPI	6	7	36.8 ± 13.5	4/11 (36.7)	HIV (1)
EPH	4	5	39 ± 15.64	3/6 (50.0)	HIV (1)
ENI	27	20	47.04 ± 12.7	4/45 (8.9)	HIV (1), HCV (1)
ENH	9	20	44.7 ± 12.4	3/27 (11.1)	HIV (6),
Total	53	60	44.6 ± 14.4	14/113	HCV (2), HIV (9)

*Nine individuals did not provide information on HBV treatment. *EPI* HBeAg-positive chronic infection, *EPH*, HBeAg-positive chronic hepatitis, *ENI* HBeAg-negative chronic infection, *ENH* HBeAg-negative chronic hepatitis.

### Serological tests

From acute infected individuals, 14/15 (93.3%) were anti-HBc total (+), 10/15 (66.7%) were anti-HBc IgM (+), 8/15 (53.3%) were HBeAg (+), 9/15 (60%) were anti-HBe (+). Two samples presented concurrent HBe/anti-HBe (+). No individual was positive for anti-HBs. Regarding coinfections, one sample was anti-HCV (+).

From chronically infected individuals, 95/98 (96.9%) were anti-HBc total (+), 10/98 (10.2%) were anti-HBc IgM (+), 4/98 (4.1%) were anti-HBs (+), 22/98 (22.4%) were HBeAg (+), 72/98 (73.5%) were anti-HBe (+). Five samples presented concurrent HBe/anti-HBe (+). No sample tested positive for HDV, two samples were anti-HCV (+), 9 samples tested anti-HIV (+).

### Molecular tests and genotyping

Median HBV viral load was 3.320 log IU/mL (min: 2.370, max: 9.0000) for acute and 3.946 log IU/mL (min: 1.709–max: 9.500) for chronically infected subjects. Groups EPI and ENI presented, respectively, the highest and lowest viral loads (Table 2).

**Table 2:** Molecular features of HBV carriers according to infection phase.

	Acute	EPI	EPH	ENI	ENH
Genotypes					
A1	7	7	6	26	21
A2	4	3	2	5	3
D1	0	0	0	2	0
D2	0	0	0	4	0
D3	2	1	1	4	4
D4	0	0	0	2	0
E	0	1	0	0	0
F2	2	1	0	4	1
S gene mutations					
< 2	6 (40%)	13 (100%)	8 (88.8%)	38 (80.8%)	21
≥ 2	9 (60%)	0	1 (11.2%)	9 (11.1%)	7
POL mutations					
< 2	2 (13.4%)	4 (30.7%)	2 (22.2%)	0	1 (3.45%)
≥ 2	13 (86.6%)	9 (69.2%)	7 (77.8%)	47 (100%)	28 (96.55%)
Mean viral load (log IU/mL)	4.6 ± 3.62	7.23 ± 1.73	3.55 ± 4.70	2.22 ± 0.8	3.2 ± 0.5

*POL* Polymerase; *EPI* HBeAg-positive chronic infection; *EPH* HBeAg-positive chronic hepatitis; *ENI* HBeAg-negative chronic infection; *ENH* HBeAg-negative chronic hepatitis.

Four HBV genotypes were found in the sampling: HBV/A (74.3%; 84/113), HBV/D (17.7%; 20/113), HBV/E (0.9%; 1/113), and HBV/F (7.1%; 8/113). Regarding subgenotype distribution, among 84 individuals infected with HBV/A, 79.7% were classified as subgenotype A1 and 20.3% as subgenotype A2. HBV/D isolates were classified as subgenotypes D2 (20%), D3 (60%) and D4 (10%). All 8 HDV/F isolates found were classified as subgenotype F2. HBV genotype distribution according to disease phases is shown in Table 2. As expected, HBV/A1 was the most prevalent genotype irrespective of CHB phase. The ENI group had the highest number of non-A subgenotype isolates, however genotypic distribution was not statistically significant among the groups (*p* = 0.42). The sequences were submitted to GenBank under the accession numbers PP439846–PP439953.

### Mutations

#### Clinically relevant mutations

Clinically relevant mutations were observed in 14 samples, 2 from acute and 12 from chronically infected individuals. The mutation sY100C in HBsAg was observed in 11 individuals, two acute and 9 chronically infected (one EPI, three EPH, two ENI and three ENH). Furthermore, at least one of the mutations sT118A/M, sM133T, sD144A and sG145Rwere observed in 6 chronically infected individuals (two ENI, two ENH and two EPI). One individual under antiviral therapy from the ENH group presented the double resistance mutation rtL180M/M204V in the RT domain of the viral polymerase.

#### HBV genetic variability

Genetic divergence analyzes were performed on sequences of the major genotype found in our sampling, HBV/A. In addition, coinfected patients were also excluded from this analyzes.

**Figure 1:**
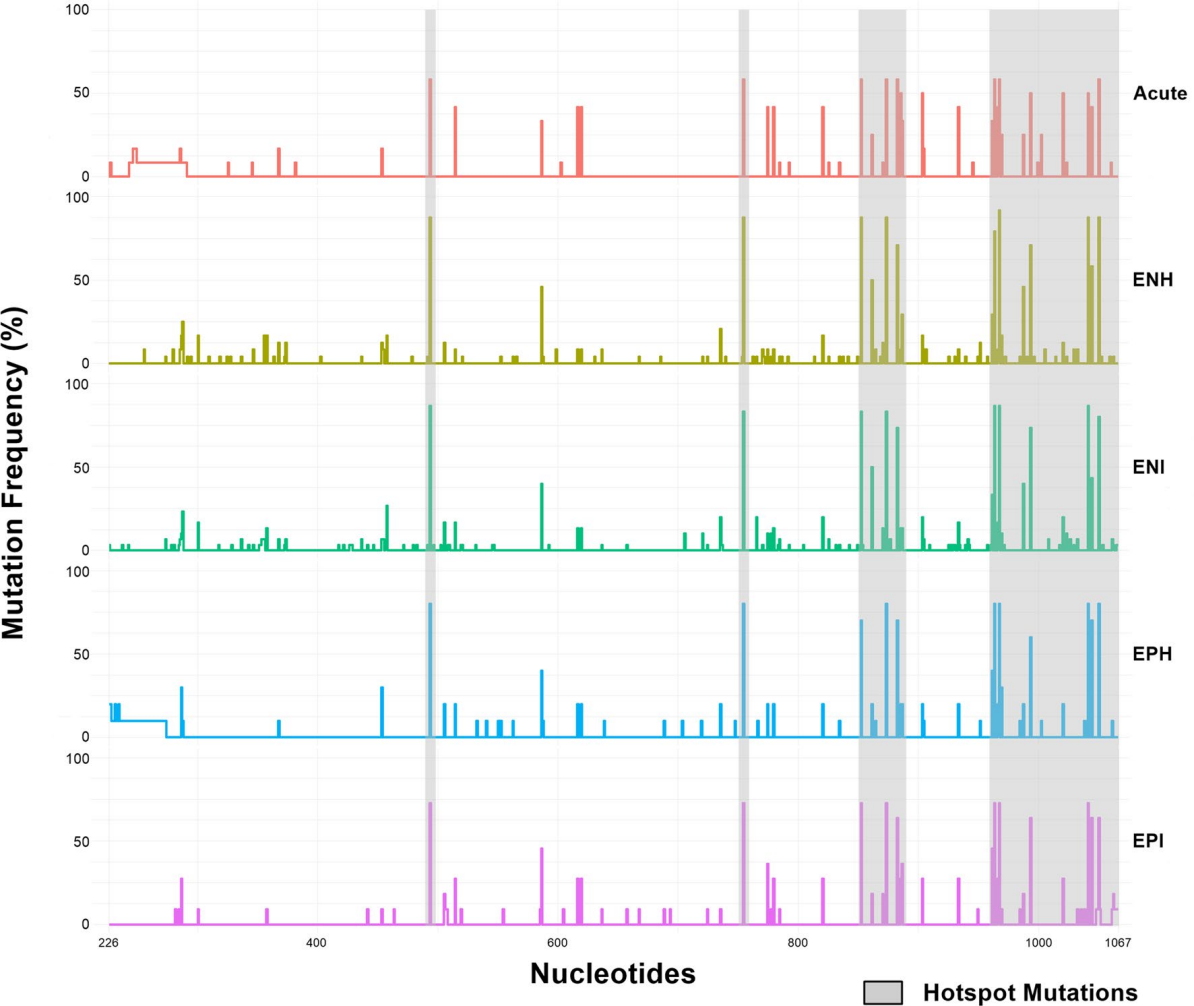
Nucleotide diversity of HBV infected patients in distinct phases of chronic and acute infection. The shaded regions are hotspot mutation zones shared by all the groups. Percentages refer to the frequency of nucleotide substitutions, adjusted by the number of sequences in each group, following the formula (Number of nucleotide substitutions × 100) ÷ total number of sequences in each group).The numbering on the X-axis corresponds to the nucleotide positions in the complete HBV genome.

As presented in Fig. 1, despite hotspot regions were similar in all chronic disease phases, differences in nucleotide frequencies can be observed. Notably, CHB HBeAg (+) groups (EPI and EPH) presented lower nucleotide diversity in the first third of the sequenced fragment compared to CHB HBeAg (−) groups (ENI and ENH).

**Figure 2:**
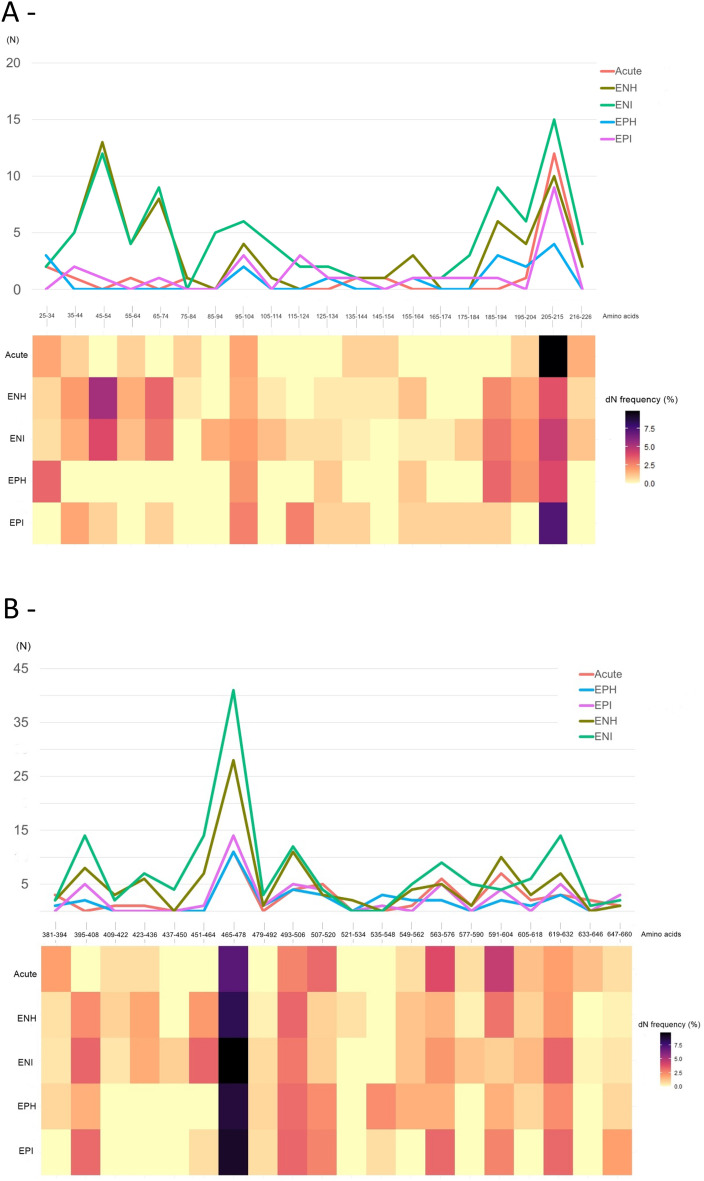
Genetic diversity of HBsAg (**A**) and Polymerase (**B**) from HBV-A infected patients in distinct acute and chronic phases. N: Number of nonsynonymous (NS) mutations per block of 10 (**A**) and 14 (**B**) amino acids (aa). Percentages refer to the frequency of NS mutations per block of amino acids, adjusted by the number of sequences in each group, following the formula (Number of NS mutations × 100) ÷ total number of sequences in each group). The X-axis indicates amino acid positions in (**A**) S gene and (**B**) polymerase. The Y-axis indicates the number of NS mutations at each position.

In addition to the higher genetic diversity of the POL non-overlapped region compared to S/POL overlapped region of all sequences, an overall higher frequency of nucleotide polymorphisms was observed in CHB HBeAg (−) groups (Table 2, Fig. 1). However, considering MHR region, higher genetic variability was observed in CHB HBeAg (+) groups and in patients with acute hepatitis B. These groups displayed the highest number of polymorphisms in “a” determinant (Fig. 2).

**Figure 3:**
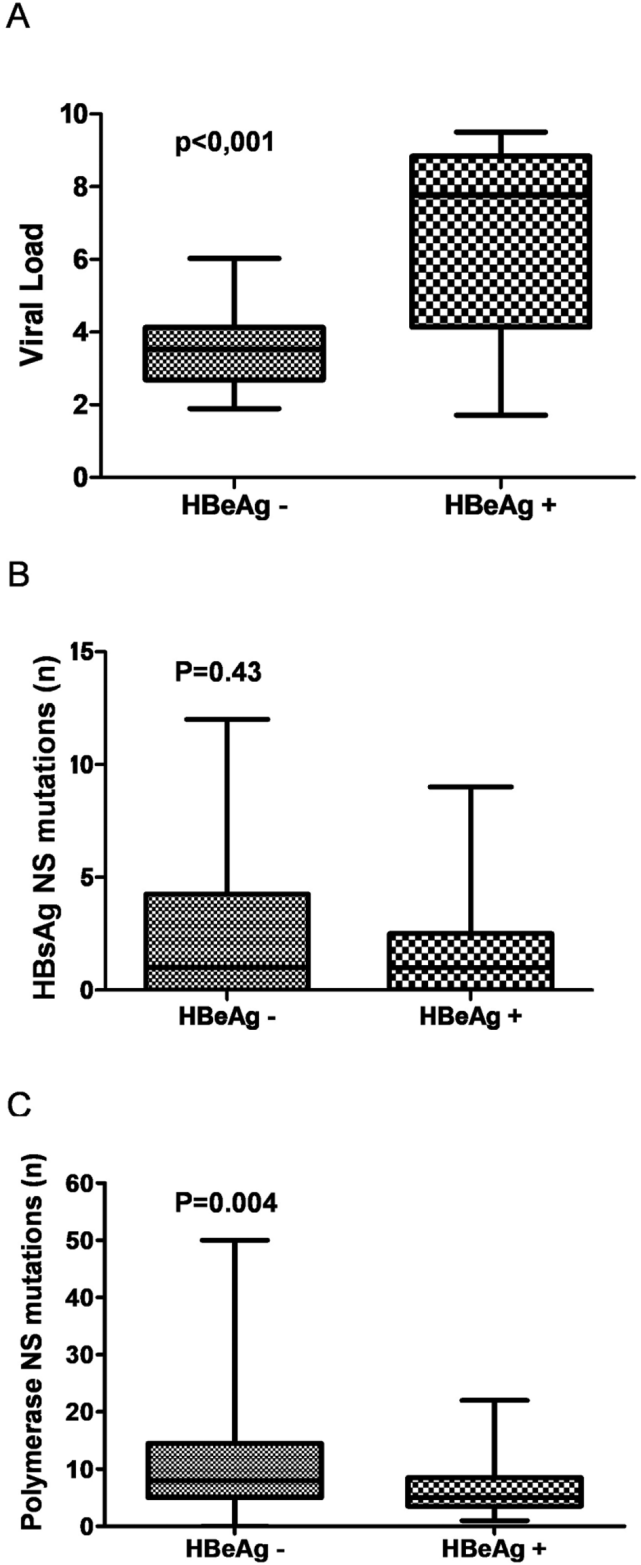
Boxplot comparing HBV loads (**A**) and HBV genetic diversity, expressed by HBsAg (**B**) and Polymerase (**C**) nonsynonymous (NS) mutations. The X-axes show HBV viral loads expressed in log IU/mL (**A**), the number of HBsAg (**B**) and polymerase NS mutations (**C**). The Y-axes corresponds to HBeAg− and HBeAg+ groups. Statistical test: unpaired t test with Welch’s correction.

The ratio of dN/dS [nonsynonymous substitution rate (dN) divided by synonymous substitution rate (dS)] was employed to measure the selection pressure for the evolution of HBsAg and partial polymerase genes according to phases of hepatitis B infection^20,21^. According to Fig. 3, CHB HBeAg (−) groups were subjected to a higher selection pressure. As expected, CHB HBeAg (−) groups presented lower viral loads compared to CHB HBeAg (+) groups (*p* < 0.001; Fig. 3A). In contrast, CHB HBeAg (−) groups displayed higher genetic diversity, expressed by the number of nonsynonymous (NS) mutations in polymerase (*p* = 0.004; Fig. 3C).

In the reverse transcriptase of the polymerase gene, the positions rtN122H/L/Y, rtY126H/C, rtL129M, rtW153R, rtI163V, rtL217R, rtV253I were the most polymorphic. Regarding HBsAg, NS mutations were more frequent in the first and second cytosolic loops (CYL1—sS45L/T, sL49P/R, sS61L, sI68N/T and CYL2—sT189I,sA194V, respectively) and in transmembrane domain 4 (TMD4—sY206N, sN207S/T, sI208T, sL209V and sS210R).

## Discussion

The natural history of hepatitis B infection comprises distinct phases that may reflect the viral adaptation to the host selection pressure. In most cases, HBeAg seroconversion is considered an indicative of convalescence and is used as a pivotal endpoint of therapy. HBeAg (−) status is often followed by loss or decrease of HBsAg and serum HBV DNA, denoting viral replication control^22,23^. Consequently, HBeAg (−) patients have a favorable prognosis and are not assumed to have overt immune-mediated liver injury^11,24^.

In this study, 113 HBV carriers in distinct clinical phases were analysed. We evaluated genotype distribution and the correlation between genetic diversity and phases of hepatitis B infection.

HBV/A and HBV/D, the main genotypes circulating in Brazil, were detected in all groups, and were phylogenetically related to Brazilian samples^25^. HBV/F was present in all but EPH group and clustered with F2 sequences from Brazil ^26–28^. HBV/E was detected in one sample from EPI group and was phylogenetically related to samples from Cape Verde^29^. As extensively reported, HBV/A1 was the most prevalent subgenotype in Brazil irrespective of acute or CHB phase, followed by HBV/D3 and F2 ^25,30–33^**.**

To avoid the bias of genetic variability, only HBV/A samples were considered for genetic diversity analysis. In addition, once the presence of other virus may influence on the natural history of HBV, coinfected samples were also excluded.

As demonstrated in this study, higher HBV genetic diversity was observed in CHB HBeAg (−) groups when compared to HBeAg (+) groups (Fig. 3). Similar findings have been reported by other authors^23,34^, which suggest that individuals with higher viral variability may have a greater chance to experience spontaneous HBeAg seroconversion.

In our findings, lower viral loads and higher HBV genetic diversity were observed in HBeAg (−) individuals (Fig. 3). This trend suggests that HBV diversity may reflect the viral ability to evade the selective pressure imposed by the host immune system, although this might lead to a reduction in viral fitness. This finding agreed with other studies^23,34^ that assume that immune selection pressure to suppress viral replication would lead to the positive selection of escape mutants, mediated by an increased the viral diversity prior to HBeAg seroconversion.

Most studies focused on genetic variability within MHR of HBsAg, where potential binding sites for B and T lymphocytes are located^35,36^. In this study, clinically relevant mutations such as sY100C, sT118A/M, sM133T, sD144A and sG145R in MHR were observed in two acute and twelve chronically infected individuals. These mutations have been associated to a reduced antigenicity and immune escape^37^. Our findings, however, indicated that most polymorphic sites were located out of the MHR, in the CYL1-2 and TMD4. Despite CYL1 contains cysteines residues (Cys), such as Cys 48, 65 and 69, which are highly conserved among hepadnaviruses and are critical for HBsAg secretion, there are few essential amino acid residues in this domain^38,39^. Nonetheless, CYL1 is known to contain important T-cell epitopes (residues 28 to 51), which can be disrupted by point mutations. Regarding TMD4, it is proving to be an important domain for HBsAg stability and release from infected cells^36^. In this study, variations in amino acid positions s45, s49, s61, s68, s189 and s194 of CYL1-2 and s206, s207, s208, s209 and s210 of TMD4 were observed. These polymorphisms have been linked to reduced HBsAg secretion and the HBsAg/anti-HBs coexistence^36,40,41^.

Analyzing polymerase amino acid residues, one patient from the ENH group displayed the double resistance mutation rtL180M/M204V. In addition, substitutions in amino acids rtN122H/L/Y, rtY126H/C, rtL129M, rtW153R, rtI163V, rtL217R, rtV253I were observed in 86.7% of HBeAg (−) patients. Some of them have been described as compensatory mutations that might restore fitness in drug-resistant viral subpopulations^42^.

The main limitations of this study are the convenience sampling and the limited number of HBeAg (+) patients. In addition, the analysis of HBV genetic diversity in the different stages of infection could not be carried out for all genotypes, being restricted to genotype A. Nevertheless, to the best of our knowledge, this is the first study addressing the HBV genetic variability of the overlapped HBV polymerase/surface gene in Brazilian patients in different phases of hepatitis B infection. Our results provide information on the molecular characteristics of HBV in a diverse clinical setting, and may guide future studies with a broader sample size.

Further studies addressing the distinct presentations of HBV infection are needed. In particular, to investigate the dynamics of HBV genetic factors during the follow-up of chronic patients could increase our knowledge of the virus’s response mechanisms face of selective immunological and/or pharmacological pressures. A better understanding of how viral dynamics may be reflected in the evolution of liver disease might help to design improved therapeutic strategies in the future.

## Material and methods

### Study population

One hundred and thirteen samples from the biorepository of the Laboratory of Viral Hepatitis collected between 2011 and 2015 were enrolled in this non‐probabilistic study. Fifteen samples were from individuals with acute hepatitis B and ninety-eight from patients in different phases of chronic hepatitis B infection: 13 HBeAg-positive chronic infection (EPI), 9 HBeAg-positive chronic hepatitis (EPH), 47 HBeAg-negative chronic infection (ENI), 29 HBeAg-negative chronic hepatitis (ENH). All individuals are over 18 years and signed the written inform consent.

This study was approved by the Ethics Committee of the Oswaldo Cruz Foundation (number 661.187/CAAE 18281313.4.0000.5248). All procedures were performed in accordance with the Helsinki Declaration of 1975, revised in 2008.

### Detection of HBV serological markers

Aiming to confirm the previous classification of the samples into the distinct HBV phases, serum samples were re-tested by commercial enzyme immunoassays (ELISA) for HBsAg (ETI-MAK-4, Diasorin, Italy), total anti-HBc (FTE AB COREKPLUS, Diasorin, Italy), anti-HBc IgM (TSI CORE IgMK Plus, Diasorin, Italy), anti-HBs (ETI-AB-AUK-3 Diasorin, Italy) and by electrochemiluminescence for HBeAg and anti-HBe serological markers (e411 Cobas, Roche Diagnostics, USA), according to the instructions of each manufacturer. In addition, antibodies to hepatitis C virus (anti-HCV), hepatitis D virus (anti-HDV) and immunodeficiency virus (anti-HIV) were tested by commercially available immunoassays (Murex Anti-HCV, DiaSorin ETI-AB-DELTAK-2 and Murex HIV Ag/Ab Combination) to evaluate co-infections.

### Quantification of HBV viral load

All serum samples were submitted to the commercial assay COBAS® TaqMan HBV^®^ Test (Roche Diagnostics, Branchburg, NJ, USA) according to manufacturer’s instructions, to determine HBV viral loads.

### HBV DNA extraction and amplification of S/POL region

HBV DNA was extracted from serum samples using a commercial kit (High Pure Viral Nucleic Acid Kit, Roche Diagnostics, Mannhein, Germany) following manufacturer’s instructions. HBV genotyping and molecular characterization were performed by direct sequencing of the overlapped partial surface/polymerase (S/POL) region (~ 900base pairs) amplified by a semi-nested PCR assay using primers described by Mallory et al.^43^ and optimized by Villar et al.^44^ Wolf et al.^21^.

### Nucleotide sequencing and sequence analyses

PCR products were purified with QIAquick gel extraction kit (Qiagen, Hilden, Germany) and direct nucleotide sequencing reaction was done using Big Dye Terminator kit version 3.1 (Applied Biosystems, Foster City, CA, USA) with external and internal primers described by Mallory et al.^43^ and Kryazhimskiy and Plotkin^20^. Sequencing reactions were performed on ABI3730 automated sequencer (Applied Biosystems).

Assembly of each sequence, multiple sequence alignment and phylogenetic analyses were done in MEGA11 software^45^. Phylogenetic trees were reconstructed using Maximum Likelihood method with bootstrap test (1000 replicates) to assess the confidence of the output tree. Consensus sequences of each HBV isolate were submitted to a web-based tool for prediction of clinically relevant mutations (Geno2pheno [hbv] 2.0, Max-Planck-Institut für Informatik, Germany, available at http://hbv.geno2pheno.org/index.php).

### Statistical analysis

Descriptive statistical analysis was performed with calculation of means and standard deviation, with a preliminary assessment using contingency tables and respective statistics. Categorical variables were compared between HBeAg (+) and (−) groups using the chi-square test or Fisher’s exact test, and continuous variables were analyzed using the Mann–Whitney U test. HBV DNA viral load correlation with genetic diversity was calculated using Pearson correlation test. *p* value < 0.05 was considered significant. Statistical analysis was determined using GraphPad InStat 3 (GraphPad InStat Sofware, San Diego, United States) and graphs were done using MedCalcSofware v.9.6.4.0 (MedCalc Sofware, Belgium).

To prepare the Figs. 1 and 2, the counts of nucleotides and amino acids, as well as the calculations of mutation frequencies within each group, were made using Excel spreadsheets. Graphical representations of this data were made using the R Studio platform version 3.3.0 and RStudio version 2023.12.0 + 369, using the “Esquisse” package.

## Data Availability

The datasets generated and/or analysed during the current study are available in the GenBank repository, under the accession numbers PP439846–PP439953.
